# Setting the forest reference levels in the European Union: overview and challenges

**DOI:** 10.1186/s13021-021-00185-4

**Published:** 2021-07-31

**Authors:** Matteo Vizzarri, Roberto Pilli, Anu Korosuo, Viorel N. B. Blujdea, Simone Rossi, Giulia Fiorese, Raul Abad-Viñas, Rene R. Colditz, Giacomo Grassi

**Affiliations:** 1 European Commission, Joint Research Centre, Via E. Fermi, 2749, TP 26/A, 21027 Ispra, Italy; 2 European Commission, Directorate General Climate Action, Brussels, Belgium

**Keywords:** Forest reference level, Forest management, Accounting, Reporting, Climate change mitigation, Climate target

## Abstract

**Background:**

The contribution of EU forests to climate change mitigation in 2021–2025 is assessed through the Forest Reference Levels (FRLs). The FRL is a projected country-level benchmark of net greenhouse gas emissions against which the future net emissions will be compared. The FRL models the hypothetical development of EU forest carbon sink if the historical management practices were continued, taking into account age dynamics. The Member States’ FRLs have been recently adopted by the European Commission with the delegated Regulation (EU) 2021/268 amending the Regulation (EU) 2018/841. Considering the complexity of interactions between forest growth, management and carbon fluxes, there is a need to understand uncertainties linked to the FRL determination.

**Results:**

We assessed the methodologies behind the modelled FRLs and evaluated the foreseen impact of continuation of management practices and age dynamics on the near-future EU27 + UK forest carbon sink. Most of the countries implemented robust modelling approaches for simulating management practices and age dynamics within the FRL framework, but faced several challenges in ensuring consistency with historical estimates. We discuss that the projected 16% increase in harvest in 2021–2025 compared to 2000–2009, mostly attributed to age dynamics, is associated to a decline of 18% of forest sink (26% for living biomass only).

**Conclusions:**

We conclude that the FRL exercise was challenging but improved the modelling capacity and data availability at country scale. The present study contributes to increase the transparency of the implementation of forest-related EU policies and provides evidence-based support to future policy development.

**Supplementary Information:**

The online version contains supplementary material available at 10.1186/s13021-021-00185-4.

## Background

Forests play an important role in climate change mitigation [1]. In the EU27 + UK, which is the scope of this analysis, forests cover more than 37% of the total area and, along with harvested wood products (HWP), contribute to balancing about 10% of total greenhouse gas (GHG) emissions [2]. The mitigation potential from EU forests strongly depends on the balance between the biophysical capacity to absorb and release carbon during the growth process (i.e. photosynthesis and respiration), the natural mortality and the harvest of forest biomass (used as material or for energy purposes). Such mitigation potential depends not only on the current management, but also on the legacy effects of past management activities—which affect both the current age class distribution and the forest composition, on natural disturbances and on the impact of climate change [3, 4]. Additional mitigation may come from using wood to replace energy intensive material and fossil fuels (so-called substitution effects) [5]. Emissions and removals from forests are reported under the Land Use, Land-Use Change and Forestry (LULUCF) sector of the GHG inventories that EU, its Member States and the UK submit annually to the United Nations Framework Convention on Climate Change (UNFCCC) [2].

Through Regulation (EU) 2018/841 (hereafter LULUCF Regulation) [6], the LULUCF sector has been included in the EU climate target of − 40% greenhouse gases (GHG) emissions in 2030 relative to 1990^1^. The contribution of the LULUCF sector towards a target is regulated by a set of specific "accounting rules” that take into account the difficulty in identifying the impact of anthropogenic activities and of factoring out the effects of natural processes and age legacy effects [7, 8] Compliance under the LULUCF Regulation requires that the sector’s accounted emissions do not exceed the accounted removals in each Member State, and in the EU as a whole. The LULUCF Regulation lays down the accounting rules for the LULUCF sector in the EU, including managed forest land, for the periods 2021–2025 and 2026–2030. For forests, the accounting is based on the concept of Forest Reference Level (FRL), a country-specific projected benchmark, i.e. a counterfactual of net emissions from managed forest land and HWP, against which the future actual net emissions will be compared. This way, each Member State will quantify its mitigation efforts in the forest sector, and gain credits—if the reported net emissions are lower than the FRL—or debits—if the reported net emissions are higher. The FRL concept incorporates the impact of the continuation of past management practices (2000–2009) on future age-related forest dynamics. Furthermore, it excludes policy assumptions and market expectations [7], thus marking a radical change with respect to the Forest Management Reference Level (FMRL) adopted under the Kyoto Protocol. Because of this change, the FRL concept triggered an intensive debate, especially on the consideration of harvest and of the dynamics of age-related forest characteristics as main drivers of the future evolution of the forest carbon sink [9–11].

**Fig. 1:**
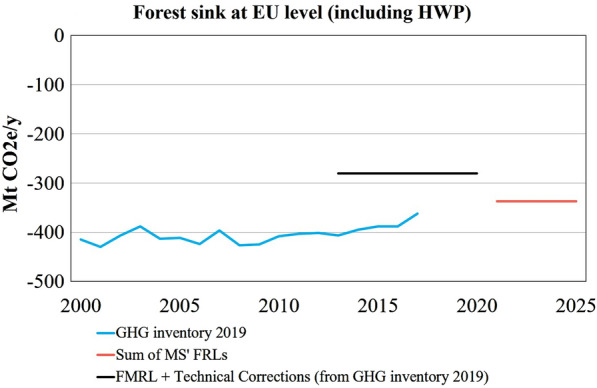
Evolution of forest carbon sink based on different information sources. Historical evolution of the EU27 + UK forest carbon sink, according to the GHG inventories, the Forest Reference Levels (FRLs), and Forest Management Reference Levels (FMRL) including technical corrections, as submitted by Member States under the Kyoto Protocol for the period 2013–2020 (https://unfccc.int/topics/land-use/workstreams/land-use--land-use-change-and-forestry-lulucf/forest-management-reference-levels). Modified from [8]

In October 2020, after a throughout process of technical assessment, the EC adopted the FRL for each EU Member State and UK for the period 2021 and 2025 [8, 12]. For this period, the projected forest sink for EU27 + UK is about 337 million tons CO_2_e year^−1^. This includes the contribution from HWP (Fig. 1), which make up to about 13% of the total sink in the FRLs (see Additional file 1: Table S1).

Recently, the EU has agreed an emission reduction of at least 55% by 2030 relative to 1990 and to reach climate neutrality by 2050 [13]. To ensure consistency with this increased climate ambition, the EC will propose revisions to the current climate legislations by mid-2021, including the LULUCF Regulation. Among the options, it has been highlighted the opportunity of a simplification of the current LULUCF rules [14, 15]. However, until this proposal becomes EU law, the current LULUCF regulation applies.

Setting FRLs is a complex exercise characterized by uncertainty. To support compliance with LULUCF regulation, a technical guidance has been developed [16]. First, FRL must be consistent with the methodological framework (i.e., data and methods) applied in current reporting of GHG emissions and removals under the UNFCCC [6]. Consistency is essential to ensuring that the future accounting of mitigation actions in managed forests genuinely reflects a deviation from past management, and not an inconsistency in methods. To demonstrate this consistency, the model used to construct the FRL must be able to reproduce historical data as reported in the GHG inventories, in particular for the reference period 2000–2009 [17]. After the first compliance period (2021–2025), the EU Member States will be required to apply technical corrections to the FRL to avoid methodological inconsistencies with the GHG inventories, possibly resulting from updates in data and methods (see also [6]). Second, the LULUCF Regulation requires Member States to ensure transparency and accuracy in the determination of the FRLs [6]. Predicting the combined impact of age-dependent growth, harvest, and mortality over time is a difficult task. Further uncertainties are associated with the model assumptions (e.g. incorporation of the effects from climate and natural disturbances) as well as with the availability of reliable data and information on forest management (harvest intensity) and age-related characteristics (increment), which should reflect country-specific circumstances [18]. Simulations have shown that, by assuming the continuation of management practices observed in 2000–2009, the EU27 + UK forest carbon sink in 2030 is expected to decrease compared to the past because of concomitant age-related impacts, a slightly constant or slightly reduced increment, and increased harvest [7]. Other studies analysed the possible economic impacts of setting limits on harvest (e.g., [9, 10]). Different assumptions, methods and approaches, including the initial age class distribution [11] may affect the outcome of such analyses for individual countries (e.g., [19]).

In this study, we explore the main challenges linked to the determination of the FRLs in the EU27 + UK, complementing and extending the currently available assessments [8, 20] with the aim to offer a scientific perspective on the FRL exercise at EU scale. In particular, by assessing the methods (data, tools and assumptions) applied by countries, we qualitatively discuss the degree of fulfilment of the main aspects covered by the LULUCF Regulation (namely the continuation of management practices, the harvest definitions and the consistency with GHG inventories), including the model adequacy and data completeness. Moreover, by quantitatively analysing the countries’ projected impact of 2000–2009 management practices and age-related dynamics on the biomass carbon sink in 2021–2025, we illustrate the main drivers behind the mitigation potential of EU forests.

## Methods

### Study area and documentation

The FRLs from individual EU Member States and UK (“countries” in the following) were submitted to the EC within revised National Forestry Accounting Plans (NFAPs) by the end of 2019, i.e. 28 documents (see Additional file 1: Table S2). In the present study, we performed a comprehensive assessment of the revised NFAPs. The assessment referred to the total area of managed forest land of about 154 million ha^2^, corresponding to about 92% of the total EU27 + UK forest area in 2018 [2]. More specifically, we carried out a full analysis of the contents of each NFAP, including: (i) the methodological approaches used to determine the FRL; (ii) the additional information as provided by some countries during 2020 concerning corrections and amendments to the NFAPs and/or the proposed FRLs (see Additional file 1: Table S2); (iii) the recalculations made by the EC leading to the adoption of FRLs at the end of 2020 [20].

### Assessment methodology

#### Assessment of the degree of fulfilment with LULUCF Regulation

We define the degree of fulfilment as a qualitative measure describing to what extent each NFAP and the associated FRL adequately reflects the relevant forest-related requirements of the LULUCF Regulation. In other words, we assessed the scientific robustness of the approaches adopted by countries towards meeting the requirements of the LULUCF Regulation, namely *what the FRL should be* (‘principles’ defined in article 8.5), *how the FRL should be determined* (‘criteria’ defined in Annex IV Part A), and *what the NFAP should contain* (‘elements’ as defined in Annex IV Part B). We categorized the degree of fulfilment into low, medium and high (see Additional file 1: Table S3). We associated each NFAP (i.e. country) to a certain degree of fulfilment for each principle, criterion and element through using specific assessment keys and guidance (see Additional file 1: Tables S3 and S4). We assessed both transparency and accuracy issues in the NFAPs linked to each item. We based our assessment of the degree of fulfilment on the guidance for building the FRLs [16], the evaluation criteria used by the EC [17, 20], and the guidelines for assessing the consistency between the FRL and the LULUCF inventories from IPCC [21]. If relevant information was missing, we used additional documentation to deepen our analysis, such as for example, publications referenced in the NFAPs. We also considered the feedbacks of the country and independent experts involved in LULUCF Expert Group meetings.^3^

For the sake of simplicity in discussing the outcomes of this assessment, we finally grouped the principles, criteria and elements into three thematic clusters (Table 1). The clusters represent a higher level of aggregation by topic: the continuation of management practices (PRACTICES—cluster 1), the incorporation/definition of harvest (HARVEST—cluster 2), and the consistency with the LULUCF inventories (LULUCF inventory—cluster 3) (see Table 1 for a more detailed description). The aggregation was obtained by summing up the number of the countries with the same degree of fulfilment (high, medium and low) for principles, criteria and elements belonging to a certain cluster.

**Table 1:** Thematic clusters and correspondence of the principles, criteria and elements in the LULUCF Regulation. Identifier and the text of the LULUCF Regulation are reported for completeness

Thematic clusters (title and brief description)	Principles	Criteria for determining the FRLs (accuracy)	Elements contained in the NFAPs (transparency of information)
Practices: Continuation of forest management practices as documented in the reference period 2000–2009	Art. 8.5 (para 1) The forest reference level shall be based on the continuation of sustainable forest management practice, as documented in the period from 2000 to 2009 with regard to dynamic age-related forest characteristics in national forests, using the best available data	Annex IV.A(b) The reference level shall ensure that the mere presence of carbon stocks is excluded from accounting Annex IV.A (c) The reference level should ensure a robust and credible accounting system that ensures that emissions and removals resulting from biomass use are properly accounted for Annex IV.A (d) The reference level shall include the carbon pool of harvested wood products, thereby providing a comparison between assuming instantaneous oxidation and applying the first-order decay function and half-life values Annex IV.A (f) The reference level should be consistent with the objective of contributing to the conservation of biodiversity and the sustainable use of natural resources, as set out in the EU forest strategy, Member States’ national forest policies, and the EU biodiversity strategy	Annex IV.B (a) A general description of the determination of the forest reference level and a description of how the criteria in this Regulation were taken into account Annex IV.B (c) A description of approaches, methods and models, including quantitative information, used in the determination of the forest reference level, consistent with the most recently submitted national inventory report, and a description of documentary information on sustainable forest management practices and intensity as well as of adopted national policies Annex IV.B (e-iii) A description of how [forest characteristics, including dynamic age-related forest characteristics, increments, rotation length and other information on forest management activities under ‘business as usual’] were considered in the determination of the forest reference level
Harvest: Dynamic age-related characteristics and management intensity	Art. 8.5 (para 2) Forest reference levels as determined in accordance with the first subparagraph shall take account of the future impact of dynamic age-related forest characteristics in order not to unduly constrain forest management intensity as a core element of sustainable forest management practice, with the aim of maintaining or strengthening long-term carbon sinks	Annex IV.A (a) The reference level shall be consistent with the goal of achieving a balance between anthropogenic emissions by sources and removals by sinks of greenhouse gases in the second half of this century, including enhancing the potential removals by ageing forest stocks that may otherwise show progressively declining sinks Annex IV.A (e) A constant ratio between solid and energy use of forest biomass as documented in the period from 2000 to 2009 shall be assumed Annex IV.A (g) The reference level shall be consistent with the national projections of anthropogenic greenhouse gas emissions by sources and removals by sinks reported under Regulation (EU) No 525/2013	Annex IV.B (d) Information on how harvesting rates are expected to develop under different policy scenarios Annex IV.B (e-iv) A description of how [historical and future harvesting rates disaggregated between energy and non-energy uses] were considered in the determination of the FRL
LULUCF Inventory: Consistency with historical estimates	Art. 8.5 (para 3) Member States shall demonstrate consistency between the methods and data used to determine the proposed forest reference level in the national forestry accounting plan and those used in the reporting for managed forest land	Annex IV.A (h) The reference level shall be consistent with greenhouse gas inventories and relevant historical data and shall be based on transparent, complete, consistent, comparable and accurate information. In particular, the model used to construct the reference level shall be able to reproduce historical data from the National Greenhouse Gas Inventory	Annex IV.B (b) Identification of the carbon pools and greenhouse gases which have been included in the forest reference level, reasons for omitting a carbon pool from the forest reference level determination, and demonstration of the consistency between the carbon pools included in the forest reference level Annex IV.B (e-i) A description of how [the area under forest management] was considered in the determination of the forest reference level Annex IV.B (e-ii) A description of how [emissions and removals from forests and harvested wood products as shown in greenhouse gas inventories and relevant historical data] were considered in the determination of the forest reference level

#### Analysis of the model adequacy

We carried out a qualitative assessment of the methodological approaches adopted by the countries for their FRL, including input data, assumptions and modelling tools. For each NFAP, we first collected information on the main characteristics (e.g. model type, scale of modelling of carbon pools, and proxies for consideration of forest age; see also [22, 23]), type of input data and covered period, incorporation of forest management practices and harvest intensity, characterization of output parameters and their consistency with the GHG inventory. Based on this information, we then assigned a certain level of adequacy, i.e. from partly adequate to highly adequate, to the modelling framework of each NFAP, and we evaluated the quality of data used as input to the modelling tools, i.e. from incomplete to complete (Table 2).

**Table 2:** Guidance table for assessing model adequacy and data quality

Main components and description (based on the theoretical approach from [52])	Covered aspects	Examples of adequacy levels
Model adequacy: Capacity of the model to simulate the development of forest carbon pools and relevant type of forest management practices and natural disturbances	Age—Simulation of age-related forest characteristics	Ability of the model to incorporate age-related proxies: Highly adequate—explicit run of age or other maturity-related parameters (individual tree size, volume classes, biomass density classes) Adequate—implicit run of age-class based on aggregated data reported in the historical GHG inventory, which assumes that age-structure would not change Partly adequate—a constant value is used
	Management—Simulation of forest management practices, and natural disturbances	Consideration of harvest intensity: Highly adequate—narrow specifications of thinning and final cuts as explicit characteristics of forest management practices and natural disturbances Adequate—broad specifications of thinning and final cuts as explicit characteristics of forest management practices, and proxies for natural disturbances Partly adequate—implicit consideration of management activities on thinning and final cuts. Natural disturbances not considered
	Pools—Incorporation of forest carbon pools	Forest carbon pools as included in the modelling approach: from mandatory pools in the LULUCF Regulation (i.e. living biomass and deadwood) [model highly adequate] to only one pool [model partly adequate] Highly adequate—modelling of C stocks and transfers among pools at disaggregated level in spatial and temporal terms Adequate—modelling of C stocks and transfers among pools at an aggregated level in spatial terms Partly adequate—multiple and non-integrated modelling framework used for simulations of each C pools (e.g. simplified models)
Data quality: Consistency of the input data	Type and quality of the input data	Use of relevant data and information sources and period matching the period 2000–2009, and consistency of data with model’s requirement (are any other assumptions made, how strong effect those assumptions are expected to have) Complete—data retrieved corresponds to modelling needs and reflects status and dynamic of anthropogenic intervention and natural disturbances in the forests Partly complete—part of the data needs to be gap filled and reconstructed based on available data Incomplete—data is missing so only expert assumptions are used as a proxy to obtain the required information on forest status and dynamic

### Statistical analysis

We implemented the Fisher’s exact test in R [24] to test the significance of the differences (*p* < 0.05) of the degree of fulfilment among clusters and individually among principles, criteria and elements (Table 1), and among adequacy types (Table 2). In other words, we aimed to evaluate whether the degrees of fulfilment and model adequacies would be significantly different among clusters (and in more detail, among principles, criteria and elements) and among adequacy types. This way, we also ex-post validated the outcomes of our assessments of degree of fulfilment and model adequacy. The Fisher’s exact test is indeed commonly used to test the independence of two nominal variables (in our case, e.g. clusters) and is more suitable than the chi-square test for smaller samples [25]. The Fisher’s exact test was also successfully used in several studies involving qualitative assessments related to forest management and biodiversity in Europe (e.g. [26, 27]).

We applied the Fisher’s exact test to two contingency tables: the first table reporting the frequency of countries associated with high, medium and low degree of fulfilment including information not available (rows) by cluster (columns; PRACTICES, HARVEST, and LULUCF inventory)—four rows × three columns; the second table reporting the frequency of countries associated with highly adequate, adequate, and partly adequate modelling approach (rows) by adequacy type (columns; AGE, MANAGEMENT, and POOLS)—three rows × three columns. We also applied the Fisher’s exact test to paired frequencies of countries by degree of fulfilment (rows) and individual principles, criteria and elements (columns; e.g. criterion $x$ vs. element $y$)—three rows × two columns.

### Quantifying the impact of management and age dynamics on future carbon sink

Harvest and natural disturbances are key drivers for the short-term development of the forest carbon sink. However, in the context of the FRL, the state of forest at the beginning of the simulation, including the age structure, might influence how the harvest intensity is defined and how the forest carbon sink develops in the future (see also [11]). We determined the impact of age-related characteristics on the future forest carbon sink through comparing the amount of CO_2_ removed by living biomass in the period 2000–2009 (i.e., living biomass carbon pool reported in countries' GHG inventories for the same period, used to define the management practices) with the simulated amount of removals in the period 2021–2025 (i.e., living biomass carbon pool reported in the NFAPs for the same period) according to equation (1):

1 $$\Delta LB=\frac{{LB}_{2021-2025}-{LB}_{2000-2009}}{{LB}_{2000-2009}}$$

where $\Delta LB$ is the variation of the living biomass carbon between the two periods, i.e. 2000–2009 and 2021–2025 (%); ${LB}_{2000-2009}$ is the reported average net carbon emissions in living biomass in the period 2000–2009 (CO_2_ ha^−1^) (source: Common Reporting Format Tables for individual countries, reporting years as in NFAPs; see Additional file 1: Table S2 and [8, 20]); ${LB}_{2021-2025}$ is the projected average of net carbon emissions in living biomass in the period 2021–2025 (CO_2_ ha^−1^) (source: NFAPs; see Additional file 1: Table S2).

Among the various forest carbon pools, we considered only living biomass as it is the only one directly removing carbon from the atmosphere and directly affected by management practices and natural disturbances. The foreseen interaction between the practices defined by countries in the period 2000–2009 and the future age dynamics result in the expected amount of harvest in the period 2021–2025. Harvest difference among the two considered periods is calculated through equation (2):

2 $$\Delta H=\frac{{H}_{2021-2025}-{H}_{2000-2009}}{{H}_{2000-2009}}$$

where $\Delta H$ is the variation of harvest amount between the two periods, i.e. 2000–2009 and 2021–2025 (%); ${H}_{2000-2009}$ is the reported average harvest amount in the period 2000–2009 (m^3^ ha^−1^) (source: NFAPs and further data provided by countries, see [8]; see Additional file 1: Table S2); ${H}_{2021-2025}$ is the projected average harvest amount in the period 2021-2025 (m^3^ ha^−1^) (source: NFAPs and further data provided by countries, see [8]; see Additional file 1: Table S2). All values are considered over bark.

Therefore, any change in harvest between the period 2000–2009 and the period 2021–2025 might be reflected in variations of the age-dependent development of the forest carbon sink among the two considered periods. This in turn means that assessing the harvest—biomass carbon sink relationship implicitly provides the magnitude of the short-term forest mitigation potential.^4^

## Results

### Degree of fulfilment

**Fig. 2:**
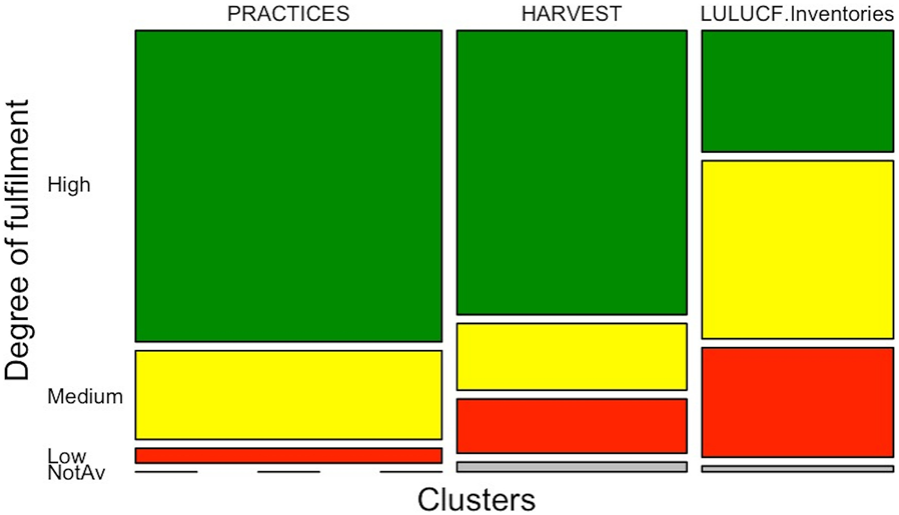
Frequency distribution of countries by degree of fulfilment (rows) and thematic cluster (columns; PRACTICES, HARVEST, LULUCF inventory; see Table 1). Columns’ width varies because of the different number of principles, criteria and elements associated with each thematic cluster (Table 1). High, medium and low degree of fulfilment are highlighted in green, yellow and red, respectively. Not available information is highlighted in grey

We found that the NFAPs and the FRLs therein mostly fulfil the requirements of the LULUCF Regulation (90% show high and medium degree of fulfilment for principles; 89% for criteria, and 82% for elements; see Fig. 2 and Additional file 1: Table S5). Countries show lower degree of fulfilment in ensuring the consistency with the GHG inventories, compared to correctly representing management practices in the period 2000–2009, and simulating the future evolution of the forest sink based on age dynamics and harvest intensity (32%, 22%, and 8% of NFAPs with high degree of fulfilment for PRACTICES, HARVEST, and LULUCF Inventory on the grand total, respectively; see Additional file 1: Table S5) (*p* < 0.05; see Additional file 1: Table S7).

Within the PRACTICES cluster, countries performed the highest degree of fulfilment in ensuring that only the changes in carbon stock are considered (and not the stock as such) in the FRL (100% of NFAPs; see Additional file 1: Table S5) (*p* < 0.05; see Additional file 1: Table S9). In general, the majority of countries ensured a consistent representation of forest management practices as in the period 2000–2009. Some countries excluded specific years, justifying this with a possible misrepresentation of the current harvest amount due to natural disturbances’ effect (e.g. Czech Republic for some years in the period 2000–2009), or lack of reliable data (e.g. Germany before 2002 and after 2007) (see also [8, 20]). These choices were however not considered to be in line with the LULUCF Regulation, and led to a recalculation of the FRL by the EC [20]. Low degrees of fulfilment are associated with the transparency in describing methods and data used in the determination of the FRL (only 54% of NFAPs with high degree of fulfilment; see Additional file 1: Table S5) (*p* < 0.05; see Additional file 1: Table S9). Most countries explicitly took into account biodiversity aspects in modelling forest management practices, usually by setting aside specific forest strata for protection or close-to-nature management (75% of NFAPs with high degree of fulfilment; see Additional file 1: Table S5) (*p* < 0.05; see Additional file 1: Table S9).

In the HARVEST cluster, countries show higher degree of fulfilment in ensuring that the simulated forest sink is based on the combination of harvest and age dynamics, compared to demonstrating that the FRL is consistent with the goal of maintaining or enhancing the forest sink over the long term (about 93% and 54% of NFAPs with high degree of fulfilment for associated principle and criterion, respectively; see Additional file 1: Table S5) (*p* < 0.05; see Additional file 1: Table S9). Countries indeed did not always provide explicit comparison between the FRL simulations and other national projections [28]. Most countries provided numerical values regarding the share of wood used for energy for the period 2000–2009 and as applied in the projections (89% of NFAPs with high degree of fulfilment; Additional file 1: see Table S5), but only half provided transparent information about the historical and future harvesting rates, disaggregated between energy and non-energy uses (50% of NFAPs with high degree of fulfilment; see Additional file 1: Table S5).

Within the LULUCF Inventory cluster, we found inconsistencies for area and pools and gases (57% and 46% NFAPs with low degree of fulfilment for related elements, respectively; see Additional file 1: Table S5). Such inconsistencies were reflected into the overarching criterion of ensuring consistency with the GHG inventory estimates, for which 92% NFAPs show only medium degree of fulfilment (see Additional file 1: Table S5) (*p* < 0.05; see Additional file 1: Table S9). We found no significant differences in terms of the degree of fulfilment about the consistency with GHG inventory between the related principle and criterion (see Additional file 1: Table S9), because they represent overlapping requirements. In detail: all countries but Malta ensured consistency in living biomass carbon pool; three countries (Croatia, Poland and Romania) incorporated deadwood in the FRL but not in the GHG inventory; nine and eight countries did not consider CO_2_ and non-CO_2_ emissions from biomass burning, respectively, although reported in the GHG inventory (see Additional file 1: Table S11). In the case of wildfires, six countries (Ireland, Italy, Luxembourg, Hungary, Portugal and the United Kingdom) reported a provisional background level for natural disturbances in their NFAPs (see [8, 20]).

### Methodological approaches to determine the FRL

**Fig. 3:**
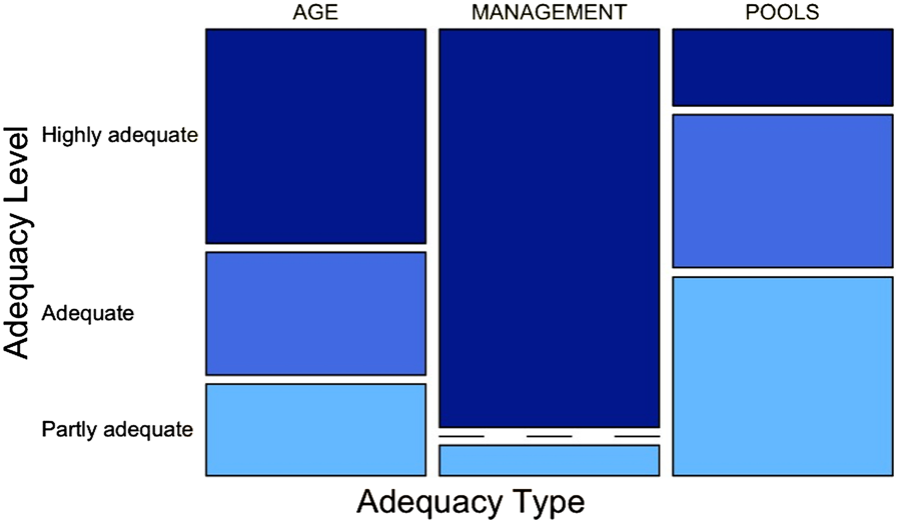
frequency distribution of countries by level of model adequacy (rows) and type (columns; AGE, MANAGEMENT, POOLS; see Table 2). Shades of blue refer to highly adequate, adequate and partly adequate modelling tools (from darker to lighter).

We found that model adequacy was higher for simulating forest management practices than for incorporating explicit age-related characteristics or additional carbon pools beyond living biomass (about 93%, 50% and 18% of countries using highly adequate models, respectively; see Fig. 3 and Additional file 1: Table S6) (*p* < 0.05; see Additional file 1: Table S8). Countries adopted heterogeneous approaches in the determination of the FRL, 24 were even different from the GHG inventories. Countries used specific modelling tools to simulate forest growth and the impact of management practices on age dynamics (see Additional file 1: Table S10). Sixteen countries adopted already existing modelling tools, eight countries developed ad hoc FRL models specifically for this exercise, and four countries implemented an IPCC methodology for carbon emissions and removals (gain-loss or stock-change method on aggregated estimates from the GHG inventory) complemented with ancillary information (see Additional file 1: Table S10).

Modelling tools are mostly parameterized with field data, and mainly focused on forest strata (e.g. forest types, species cohorts or main species; see Additional file 1: Table S10). This way, countries were able to stratify the managed forest land and assign forest management practices to each stratum. Depending on the modelling tool, the age and age-related forest characteristics were defined differently among countries (see Additional file 1: Table S10). Half of countries considered age as an explicit input (e.g. stand age), and half of countries adopted alternatives such as age-related proxies (e.g. tree dimension/size) or other parameters (e.g. volume classes, biomass density). Harvest intensity, as the core quantitative element of defining the forest management practices, was set in terms of: harvest volume per growing stock (13 countries); harvest probabilities per strata or age class (6 countries); harvest area per area available for harvest combined with additional elements (3 countries); harvest per increment (3 countries); harvest volume per hectare (2 countries); no harvest (1 country) (see Additional file 1: Table S10). The resulting outcome parameters (age-related forest characteristics) mainly regard area and volume (35% and 26% of cases, respectively; see Additional file 1: Table S10).

Countries used complete or partly complete input data for the modelling exercise (18 and 10 countries, respectively). For the definition of data completeness, please refer to Table 2. Data completeness was found higher for Western European countries (covering about 26% of the total managed forest land) compared to Central and Eastern European countries (about 19% of the total forest area), and higher for Northern European countries (covering more than 37% of the total forest area) compared to Southern European countries (covering about 18% of the total forest area).^5^ Apart from the regional assemblage, it should be pointed out that data completeness also refers to the transparency of the information reported into the NFAPs as well as to the overlap of input data with the period 2000–2009. The main sources of information for input data were forest inventories directly (35% of cases), followed by GHG inventory databases (23% of cases) and other forestry statistics (18% of cases) (see Additional file 1: Table S10). Of course, some of these information sources are not mutually exclusive, since National Forest Inventories (NFIs) and national statistics actually feed the background data for the GHG inventories [2]. Countries used complementing information from technical reports/scientific studies/expert judgments/questionnaires (15% of cases), or from regional and local forest management plans (9% of cases) (see Additional file 1: Table S10).

### Evolution of living biomass carbon sink and change in harvest level

**Fig. 4:**
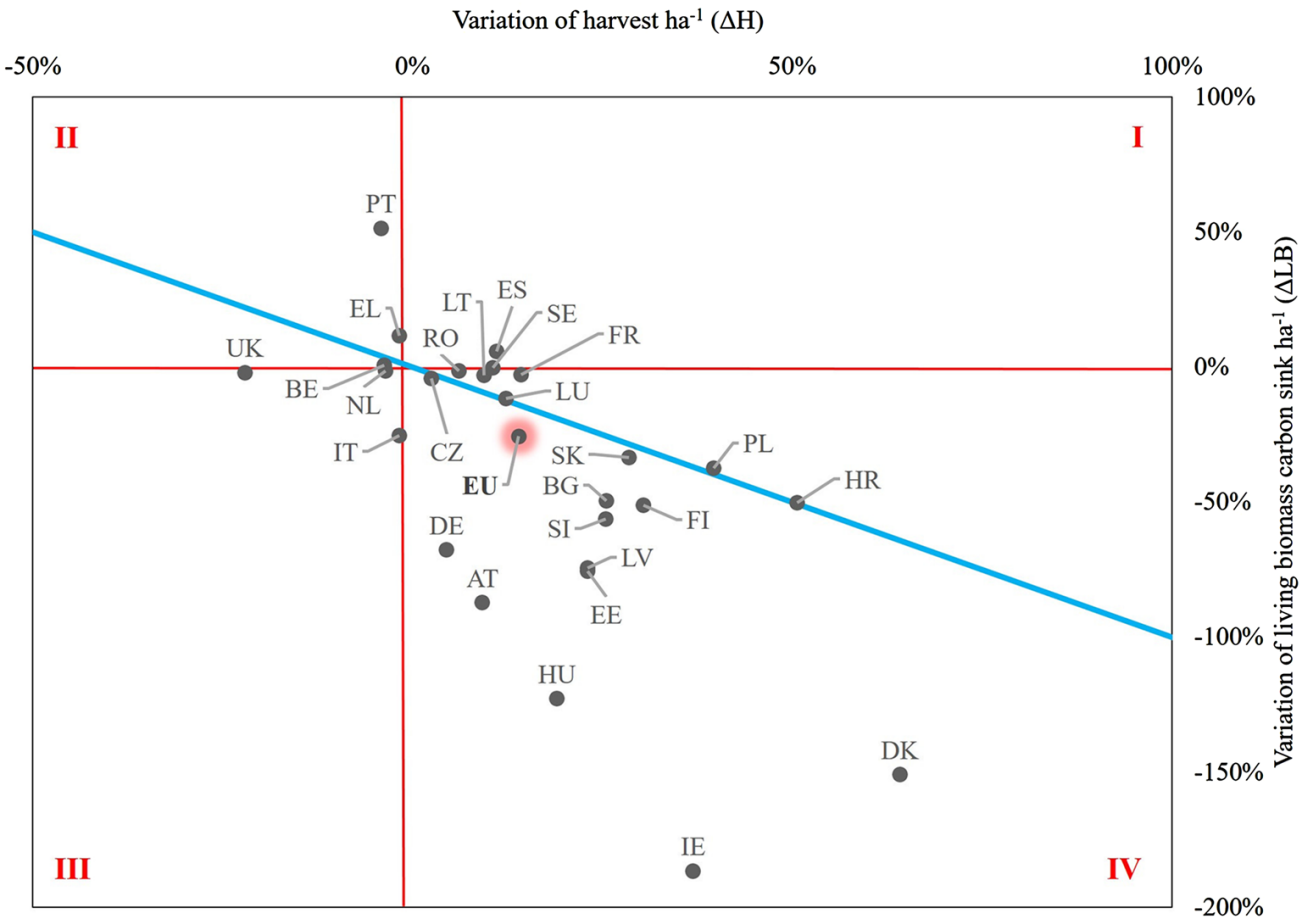
Correlation between changes in harvest and changes in living biomass carbon sink. Black dots by country indicate the changes in the living biomass carbon sink and in the harvest amount, between the period 2000–2009 and the period 2021–2025, both scaled on area unit. EU25 + UK (excluding Cyprus and Malta) is highlighted in red. Solid blue line is the 1:1 line of harvest changes from the period 2000–2009 to the period 2021–2025. Quadrant I: increased harvest—increased sink; quadrant II: reduced harvest—increased sink; quadrant III: reduced harvest—reduced sink; quadrant IV: increased harvest—reduced sink

Figure 4 reports the relative variation in the living biomass carbon sink and in the amount of harvest between the periods 2000–2009 and 2021–2025. Results show that the overall carbon sink in living biomass per hectare in EU25+UK—i.e. the sum of individual countries’ estimates, excluding Cyprus and Malta—decreases by about 26% in the period 2021–2025 compared to the period 2000–2009, with a corresponding 14% increase of the amount of harvest per hectare. These estimates are slightly different than those reported in [8] because we considered the living biomass only, further scaled per unit area, while [8] provide results including all pools.

Nineteen out of 26 countries (no data available for Malta and Cyprus) simulated a decreasing carbon sink and an increasing amount of harvest for the period 2021–2025 compared to 2000–2009 (see quadrant IV in Fig. 4). For most of these countries, the age class distribution follows a normal (12 countries, and all countries together) or a reverse-J (9 countries) shape (see Additional file 2: Figure S1), allowing for an increasing amount of harvest within the period 2021–2025. For most of these countries, the relative reduction of the biomass carbon sink is larger than the corresponding increase of harvest. This can be mostly attributed to the ongoing aging process, resulting in a progressive decrease of net annual increment and an increase of mortality.

An increasing amount of harvest has no direct effect on the biomass carbon sink in the period 2021–2025 for five countries (zero line between quadrants I and IV in Fig. 4). This is the case for example of France, Lithuania and Sweden, or even of Spain where forests mostly have an uneven-age structure (see Additional file 2: Figure S1). Where the age class distribution is quite uniform (i.e., for Czech Republic) or irregular, the amount of harvest within the period 2021–2025 was probably determined by other variables (e.g., salvage logging after disturbance events) rather than the shape of the age class distribution. Six countries simulated a slightly decreasing or stable amount of harvest in the period 2021–2025 (see quadrants II and III in Fig. 4). Despite this reduction, UK, Belgium and the Netherlands projected a stable biomass sink. This may suggest that the amount of harvest decreases proportionally to the net annual increment. Greece and Portugal estimated an increasing carbon sink, which could be attributed to an increasing current annual increment. Italy projects that a reduction of the current annual increment—due to the ongoing aging of the forests—causes a decline in the future living biomass carbon sink (see also [29]). In this case (as also for e.g. Greece), the age class distribution was not the key driver in determining the future amount of harvest because forests mostly have an uneven-aged structure. In other cases (e.g. Croatia, Poland), an increase in harvest would result in a proportional decrease of forest carbon sink in the next years (Fig. 4).

## Discussion

### Facing the modelling complexity

The FRL is an outcome of complex modelling exercise performed individually by each country. Based on our assessment, the main difficulty faced by countries was ensuring the consistency between the FRL and GHG inventories, or with other estimates (e.g. national GHG emission projections^6^) (see Fig. 2 and Additional file 1: Table S5). Compared to the FMRL under the Kyoto Protocol, where the amount of harvest was implemented as an exogenous driver within the modelling framework (including economy or policy assumptions), the FRL concept is more difficult to implement because in principle it requires forest models to simulate management practices, age dynamics and the resulting harvest endogenously (cf. [30]; see also Fig. 1). The majority of countries adopted advanced forest ecosystem models, while some needed to develop ad hoc FRL models to comply with the requirements of the LULUCF Regulation (see Additional file 1: Table S10).

Additional challenges derived from country capacities, in terms of data, know-how, and resources availability. In some cases, the limited availability of more detailed data on forest management practices, ecosystem health and economics, might affect the advancements in forest modelling (e.g. [31–33]). Driven by international commitments and an increasing interest on climate change mitigation and adaptation policies, the overall capacity for forest modelling has constantly increased in recent years (e.g. [34]). For the purposes of the FRL, the majority of countries adopted empirical models mostly based on data and information from NFIs (see Additional file 1: Table S10). Despite NFIs are conceived as the most reliable information source for forest state and management [16, 35], usually over periods (inventory cycles, every 5–10 years), such information is not always comparable between subsequent periods (see e.g. [36]). Moreover, not all countries have full matching between inventory cycles and the period 2000–2009 (e.g. Poland; see Additional file 1: Table S10).

Based on our assessment, several countries faced difficulties in collecting reliable information to adequately represent the forest management practices in the period 2000–2009. To overcome this issue, countries adopted very different approaches for quantifying the impact of forest management practices, i.e. harvest intensity, and used aggregated data or ancillary information along with NFIs (see Additional file 1: Table S10).

Other major challenges are linked to the consideration of age-related forest characteristics, including the simulation of their dynamics. In our analysis, we refer to “age” as an explicit model parameter, but we recognise that other dynamic parameters (e.g. DBH, biomass density) might be also used to adequately simulate the development of forest stands. Indeed, some countries (e.g. Germany, Italy, Portugal) used age-related proxies, such as biomass densities, volume classes or area-based increment (see Additional file 1: Table S10). Individual choices of the best proxies for age dynamics were likely driven by biophysical circumstances, data availability and parameters in statistics at country scale. The use of age “as is” can be meaningless in complex structures, such as for example, uneven-aged, or multi-layered stands in Mediterranean forests (e.g. [37]).

These findings reveal that, while countries demonstrated huge efforts in data collection and elaboration, a further improvement of data on forest management (on practices, target species, rotation length or tree cutting characteristics, harvesting rates) and characteristics (age structure, area, increment, health status, soil conditions, regeneration, etc.) would enable more robust comparison between past and future management practices, and ultimately support the decision-making process. Further harmonization of NFIs, i.e. common definition of key parameters and data processing procedures, may be an effective solution to improve comparability of forest indicators and estimates among countries [38]. Several attempts to harmonizing NFI data have been made so far, such as for example, those concerning the assumptions and definitions of stem volume [35], and of the area restrictions to forest management [39]. An improvement of NFIs should also aim at a more holistic knowledge of forests, as forest data is used for other purposes than wood resources, including climate, energy and biodiversity in the context of current policy settings. The use of remote sensing techniques, if duly combined with ground plots, will increasingly complement country statistics in providing timely spatial and temporal patterns on forest management [40, 41]. Additional efforts can be oriented to improving the robustness of national forest statistics and implicitly their reporting within the EU frameworks (e.g. EUROSTAT) or at a broader scale (e.g. FAOSTAT, Forest Europe) (e.g. [34, 42, 43]). Joint efforts aimed at assessing, comparing and enhancing forestry models in Europe can be conveyed into a common platform for sharing experiences, ideas and main findings (e.g. community of practice on forest management decision support systems; see Footnote 6).

### Ensuring consistency and comparability with historical estimates

The LULUCF Regulation requires ensuring consistency between the FRL-related simulations and GHG inventories. The reason is twofold: the accounting will be based on the GHG inventories and historical estimates presented by GHG inventories are subject to accurate and robust review process. Medium and low fulfilment in ensuring consistency with GHG inventories can be partly explained by only limited model adequacy (e.g. about pools and gases) (see Fig. 3 and Additional file 1: Table S8). The main challenges are linked to the difficulties in transitioning from simplified methods used in GHG inventories (i.e. few strata) to an increased modelling complexity for simulating the impact of past harvest and age structure development, as required by the LULUCF Regulation. This is particularly the case of modelling living biomass, for which countries further developed their modelling capacity through adopting specific modelling tools and collecting/refining detailed country-specific data (see Additional file 1: Table S10; see also the approaches used in the GHG inventories^7^).

From our assessment, the majority of countries put efforts in modelling living biomass and HWP carbon pools, and only partly deadwood, and often omitted the CO_2_ and non-CO_2_ emissions linked to biomass burning (i.e. controlled burning and wildfires), thus triggering an obvious inconsistency with the GHG inventories (see Additional file 1: Table S11). For HWP, all countries used the “production approach” following the IPCC guidelines and as required by the LULUCF Regulation (see also [8]), so consistency with the GHG inventory was not a concern.

The omission, notably of non-CO_2_ emissions, from biomass burning (prescribed and wildfires) may be due to the fact that they were considered negligible in the reference period 2000–2009, by Northern countries [2], or are to be included later using the background level for applying the natural disturbance provision, particularly concerning the fire-prone countries (e.g. Greece). Depending on the model used, countries faced difficulties in incorporating the deadwood pool (mandatory for the LULUCF Regulation), likely because of the lack of reliable data (some GHG inventories lack estimations for this pool and instead assume the pool to be in balance) (cf. [20]). In addition, many countries did not incorporate other carbon pools such as litter and soil (see Additional file 1: Table S11). This performance outcome is closely linked to the adopted modelling framework (from simplified to full carbon models), and associated data requirements. On the one hand, empirical models running exclusively aboveground biomass growth (see Additional file 1: Table S10), which are robust in simulating stand productivity, are often not able to represent carbon and nutrient cycles in other C pools, below-ground processes, and the impact of environmental disturbances (see e.g. [44]). On the other hand, widely tested models, i.e. through years of application for forestry operations and for scientific purposes at national scale or in international contexts, were used by some countries, including EFISCEN Space by Netherlands; CBM by Czech Republic, Ireland and Poland for living biomass; and Yasso by Austria, Finland, Germany and Latvia for soils (see Additional file 1: Table S10). However, the use of an advanced modelling tool providing full carbon simulations (i.e. comprising living biomass, dead organic matter and soil) made it difficult to ensure a consistent representation of all carbon fluxes as reported in the GHG inventories (e.g. Poland and Czech Republic). This is also due to different data processing and aggregation to national scale, and models’ capacity to represent the disturbances and management practices, compared to the simplified assumptions as in GHG inventories. Ensuring consistency with other information sources (i.e. time series in GHG inventory) requires additional efforts for model calibration and validation in order to improve model robustness and reduce uncertainty, such as e.g., adequate representativeness of forest diversity, accuracy of allometric equations, spatial extrapolation of local data, and conversions from standing volume to entire carbon stocks [45]. To our knowledge, six countries showed an inconsistency in the model output [20]. Three of them (Greece, France and Finland) smoothed this discrepancy by adopting an ex-post calibration, while for the remaining (Cyprus, Bulgaria, Germany), the EC put forward a correction of the FRL value because of a detected inconsistency of model outputs with GHG inventory estimates [8, 20].

The improvement of comparability between FRL and GHG inventories would require a further development of forest ecosystem models to feed both GHG inventory data and projections towards robustly incorporating both reliable input data and representation of the effects of management and environmental disturbances on stand development and growth (e.g. forest landscape models; [46]). Based on our findings, the FRL exercise resulted in an increased availability of updated data and previously disclosed information on forest management within the NFAPs, particularly on harvest [8]. These data may facilitate for example, the effectiveness of the EU-level forest initiatives (e.g. the Forest Information System for Europe—FISE,^8^ the EU forest observatory, the ThinkForest^9^ platform) in providing timely evidence-based support to current EU policies also beyond climate [47]. Advances in modelling approaches and data quality may also improve the reporting of GHG emissions and removals for forest land under the UNFCCC, and foster the comparability of estimates within the LULUCF sector [8].

### FRLs as a tool for understanding the mitigation potential of EU forests

The FRL represents the projected evolution of the forest sink (including HWP) for the period 2021–2025, with the assumption of continuing the 2000–2009 management practices and without external influences from policy and market development. This way, the FRL is a benchmark for measuring the climate impact of management changes in forestry—but it is important to note that the FRL is not a projection of probable or preferable development of the carbon sink for the period 2021–2025 (Fig. 1). The trend of the total EU forest carbon sink under the FRL (− 18% in 2021–2025 relative to 2000–2009) can be largely attributed to (i) the impact of increased harvest rates (+ 16%; see [8]) driven by the evolution of the age class distribution; and (ii) the effects of forest aging on reduced increment ([48, 49]).

The link between the age class distribution and the evolution of harvest within the period 2021–2025 is evident where even-aged forests are predominant, and harvest is mostly provided through clear-cuts. In most of these cases, the overall shape of the age class distribution confirms that the increasing amount of harvest reported within the period 2021–2025 is mostly due to the expected evolution of the age structure [11]. In other cases, however, where an irregular or an uneven-aged structure is predominant, and harvest is mostly provided through thinnings or single-tree selection systems, age structure does not play a key role. This is, for example, the case of Spain and Greece, where most of the forest area is classified as uneven-aged. In other cases, the effect of exceptional natural disturbances affecting some countries within the period 2000–2009 (i.e. Germany or Austria) or during the most recent years (such as in case of Czech Republic) might have altered the age class distribution. In these cases, salvage logging activities—which do not have a direct relation with the age class distribution—may prevail on ordinary management practices carried out within the period 2000–2009. The current FRL design tried to balance the impact of all these factors—certainly having different roles due to country-specific circumstances—and, at the same time, factored out possible expectations due to policy and economic assumptions, allowed under the Kyoto Protocol [7].

Our analysis suggests that the projected carbon sink in living biomass decreases more than proportionally (− 26%) compared to the increasing amount of harvest (+14%) projected in the FRLs (Fig. 4). Since this sink is the difference between net increment and harvest, when most of the increment is harvested, then a relatively small increase in harvest causes a significant drop in the sink. For example, if the increment is 100 tC, the harvest 80 tC and the sink is 20 tC, a 10% increase in harvest (88 tC) causes a 40% drop in the sink (from 20 tC to 12 tC, assuming a constant increment). This projected trend in age-related increase in harvest calls for additional efforts in order to reverse the current declining sink and align the forest sector with the mitigation expected in 2030. On the one hand, an urgent increase in net increment would be required [5], e.g. through new forest area or improved forest management practices (thinning etc.). On the other hand, a climate-smarter use of any extra age-related harvest becomes even more important, i.e. using this extra wood in long-lasting products with high material substitution benefits may partially compensate the impact of the declining forest sink [5].

## Limitations of the study

As any other qualitative analysis, our assessment of the degree of fulfilment is partially based on expert judgment. Subjectivity might be introduced because of the different level of knowledge and type of the experts involved in the assessment (e.g. [50]). We ensured a certain robustness in our assessment through making best use of the guidance documents and considering the most relevant feedbacks from country and independent experts working in the process of the implementation of the LULUCF Regulation (lasting 2 years). Similar approaches have been adopted in other studies (e.g. analysis of urban forest management plans; [51]). In addition, the lack of comprehensive studies other than those already used as background information [8, 20] could have hampered a robust comparison and cross-validation of our assessment outcomes. The entire assessment derives from the information reported in the NFAPs and other relevant documents written in English, which were publicly available from and/or officially provided by countries or the EC. This choice could have excluded from the analysis additional (possibly) useful information available at country level, likely not in English.

## Conclusions

This study provides an overview of the methods and approaches used in the determination of the FRLs, and discusses the main aspects affecting the projected mitigation potential of forests in the EU. We find that ensuring consistency of FRL with the GHG inventories has been the main challenge faced by countries. We also highlight how the technical difficulties associated with the setting of FRLs made the entire process complex and lengthy, and transparency was not always fully ensured. On the other hand, the FRL exercise was useful to collect new forest-related information within the EU, improve the forest modelling capacity in some country, and increase the credibility of the post-2020 EU forest accounting compared to the Kyoto Protocol. Irrespective of the possible future modality of inclusion of LULUCF in the EU climate target, the present study contributes to a better understanding of the short-term carbon impact of continuing the recent forest management practices, offers insights on the main drivers of the forest sink and thus may help in designing forest-related climate policies. For example, in order to minimize the negative impact of the expected age-related increase in harvest on the forest sink, policies could stimulate actions to increase the net increment (e.g. new forest area or improved forest management practices) and the use of wood in long-lasting products.

## Supplementary Information

**Additional file 1.** Detailed input data and information sources; detailed assessment results for degree of fulfilment and model adequacy.

**Additional file 2.** Supplementary figure supporting the harvest-biomass sink assessment results.

## Data Availability

All data generated or analysed during this study are included in this published article and its supplementary information files. Information sources are reported in Additional file 1: Table S2 and in [8, 20]*.*

## Abbreviations

C: Carbon
CO_2_: Carbon dioxide
CO_2_e: Carbon dioxide equivalent
DBH: Diameter at Breast Height
EC: European Commission
EU: European Union
FMRL: Forest Management Reference Level
FRL: Forest Reference Level
GHG: Greenhouse gases
HWP: Harvested Wood Products
IPCC: Intergovernmental Panel on Climate Change
LULUCF: Land Use, Land Use Change and Forestry
NFAP: National Forestry Accounting Plan
NFI: National Forest Inventory
UNFCCC: United Nations Framework Convention on Climate Change

## Footnotes

1 Based on the EU 2030 climate and energy framework, available at: https://ec.europa.eu/clima/policies/strategies/2030_en#tab-0-0

2 Actual area value used for projections within the FRL framework [20].

3 More information available at: https://ec.europa.eu/transparency/expert-groups-register/screen/expert-groups/consult?do=groupDetail.groupDetail&groupID=3638&Lang=EN.

4 We do not consider the HWP pool in our calculation because of its delayed contribution to the overall forest mitigation potential over a longer time period.

5 Regional assemblages based on: https://eur-lex.europa.eu/browse/eurovoc.html?params=72,7206#arrow_7206.

6 More information available at: http://www.forestdss.org/CoP/.

7 see “Methodologies_LULUCF_Annex” within Annex III – Methodological descriptions of the EU’s 2020 GHG inventory submission under the UNFCCC, available at: https://www.eea.europa.eu/themes/climate/eu-greenhouse-gas-inventory.

8 More information available at: https://forest.eea.europa.eu/.

9 More information available at: https://efi.int/policysupport/thinkforest.
