# Supplementary material for: Setting the forest reference levels in the European Union: overview and challenges
Source: Carbon Balance Manag. 2021 Jul 31;16:23. doi: 10.1186/s13021-021-00185-4 (PMC8325867; doi:10.1186/s13021-021-00185-4)
Supplement: Supplementary file 1 — Additional file 1. Detailed input data and information sources; detailed assessment results for degree of fulfilment and model adequacy. [file 13021_2021_185_MOESM1_ESM.pdf]

Additional file 1 – Supplementary Tables

Setting the Forest Reference Levels in the European Union: overview and challenges

Matteo Vizzarri, Roberto Pilli, Anu Korosuo, Viorel NB Blujdea, Simone Rossi, Giulia Fiorese, Raul Abad-Viñas, Rene Colditz, and Giacomo Grassi

Table S1: Overview of FRLs from Member States and UK with and without HWP (average in the period 2021-2025). Values are in tonnes CO_2eq year^{-1}. 1

Table S2: List of references for revised NFAPs and addendum / corrigendum / recalculation... 2

Table S3: Description of the degrees of fulfilment of principles, criteria and elements.. 6

Table S4: Guidance table for assessing the degree of fulfilment of NFAPs to the LULUCF Regulation. 8

Table S5: Number of NFAPs meeting degrees of fulfilment for requirements (principles, criteria and elements) of the LULUCF Regulation. Colours identify different clusters (defined in Table 1): yellow for PRACTICES, blue for HARVEST, and green for LULUCF Inventory. 11

Table S6: relative distribution in the number of NFAPs according to the assigned adequacy (see Table 2). 12

Table S7: overview of p-values resulting from Fisher's exact test for the degree of fulfilment (by cluster; see Table 1)... 13

Table S8: overview of p-values resulting from Fisher's exact test for the model adequacy (by type of adequacy; see Table 2)... 14

Table S9: overview of p-values resulting from Fisher’s exact test for paired principles, criteria and elements of the LULUCF Regulation.. 15

Table S10: List of the modelling approaches used to determine the FRL and related characteristics in terms of model functioning, input and output parameters, and consistency with the approach adopted in the GHG inventories.................................... 16

Table S11: Overview of the consistency about carbon pools and CO2 and non-CO2 gases between NFAPs and GHG inventories (source: [1]). Values represent the number of countries. 24

References 26

Table S1: Overview of FRLs from Member States and UK with and without HWP (average in the period 2021-2025). Values are in tonnes CO_2eq year^{-1}.

Two-letter country code – protocol order	Country	FRL with HWP (source: [1])	FRL without HWP (source: [2])
BE	Belgium	-1,369,009	-1,235,641
BG	Bulgaria	-5,105,986	-4,808,056
CZ	Czech Republic	-6,137,189	-4,739,425
DK	Denmark	354,000	545,000
DE	Germany	-34,366,906	-26,209,877
EE	Estonia	-1,750,000	-1,330,000
IE	Ireland	112,670	1,506,091
EL	Greece	-2,337,640	-2,164,050
ES	Spain	-32,833,000	-28,971,000
FR	France	-55,399,290	-52,292,549
HR	Croatia	-4,368,000	-3,906,000
IT	Italy	-19,656,100	-19,335,400
CY	Cyprus	-155,779	-169,569
LV	Latvia	-1,709,000	-298,000
LT	Lithuania	-5,164,640	-4,455,320
LU	Luxembourg	-426,000	-413,000
HU	Hungary	-48,000	291,000
MT	Malta	-38	-38
NL	Netherlands	-1,531,397	-1,524,424
AT	Austria	-4,533,000	-1,659,000
PL	Poland	-28,400,000	-24,384,000
PT	Portugal	-11,165,000	-10,556,000
RO	Romania	-24,068,200	-21,475,600
SI	Slovenia	-3,270,200	-2,876,700
SK	Slovakia	-4,827,630	-3,661,430
FI	Finland	-29,386,695	-23,490,244
SE	Sweden	-38,721,000	-34,348,000
UK	United Kingdom	-20,701,550	-19,755,260
EU-27 + UK		-336,964,579	-291,716,492

Table S2: List of references for revised NFAPs and addendum / corrigendum / recalculation.

Country	Source for revised NFAP (links retrieved from [1])	Source for Addendum / Corrigendum / Recalculation
Belgium	https://www.cnc-nkc.be/sites/default/files/report/file/national_forestry_accounting_plan_-_belgium_-_18122019_1.pdf	
Bulgaria	https://www.moew.government.bg/en/national-forestry-accounting-plan-of-bulgaria-including-forest-reference-levels-for-the-period-2021-2025/	Recalculation (see [1])
Czech Republic	https://www.mzp.cz/C1257458002F0DC7/cz/opatreni_v_ramci_lulucf/$FILE/OEOK-CZ_NFAP_FRL_final-20200203.pdf	Recalculation (see [1])<br><br>https://www.mzp.cz/C1257458002F0DC7/cz/opatreni_v_ramci_lulucf/$FILE/OEOK-Corrigendum_to_the_Czech_NFAP-20200608.pdf
Denmark	https://en.kefm.dk/media/12970/dnfap_revised_2019_web20191219.pdf	
Germany	https://www.bmu.de/fileadmin/Daten_BMU/Download_PDF/Klimaschutz/nfap_germany_bf.pdf	Recalculation (see [1])<br><br>https://www.bmu.de/fileadmin/Daten_BMU/Download_PDF/Klimaschutz/addendum_nfap_bf.pdf
Estonia	https://www.envir.ee/sites/default/files/national_forestry_accounting_plan_2019_final.pdf	
Ireland	https://www.agriculture.gov.ie/ruralenvironmentsustainability/climatechangebioenergybiodiversity/lulucf/	https://www.agriculture.gov.ie/media/migration/ruralenvironment/climatechange/AddendumtoIrelandsNFAPandFRLApril202000420.pef (NOT WORKING)
Greece	https://ekpaa.ypeka.gr/wp-content/uploads/2020/04/NFAP_March-2020.pdf	https://ekpaa.ypeka.gr/wp-content/uploads/2020/04/Corrigendum-to-the-NFAP.pdf

Country	Source for revised NFAP (links retrieved from [1])	Source for Addendum / Corrigendum / Recalculation
Spain	https://www.miteco.gob.es/es/cambio-climatico/temas/mitigacion-politicas-y-medidas/Estrategia.aspx	
France	https://www.ecologique-solidaire.gouv.fr/sites/default/files/Plan%20Comptable%20Forestier%20France.pdf	https://www.ecologie.gouv.fr/sites/default/files/Corrigendum%20and%20Addendum%20National%20Forest%20Accounting%20Plan%20for%20France%20%202019.pdf
Croatia	https://mzoe.gov.hr/UserDocsImages//KLIMA/SZKAIZOS//december_nfap_2019.pdf	
Italy	https://www.minambiente.it/sites/default/files/archivio/allegati/clima/nfap_final_resubmission_2019_clean.pdf	
Cyprus	http://www.moa.gov.cy/moa/fd/fd.nsf/447F6A84B70700B2C2257D1C0040CB63/$file/NFAP_FRL_CYPRUS_REVISED_31DEC2019.pdf	Recalculation (see [1])
Latvia	https://www.zm.gov.lv/public/ck/files/2019_03_1_NFAP.PDF	https://www.zm.gov.lv/public/ck/files/Addendum_to_NFAP_and_FRL.pdf
Lithuania	https://am.lrv.lt/uploads/am/documents/files/KLIMATO%20KAITA/Studijos%2C%20metodinė%20medžiaga/National%20Forestry%20Accounting%20Plan%20of%20LT_revision_2020%2002%2002_submitted.pdf	
Luxembourg	https://cdr.eionet.europa.eu/lu/eu/mmr/lulucf/envxhmznq	
Hungary	http://cdr.eionet.europa.eu/hu/eu/mmr/lulucf/envxgc1ma	
Malta	http://cdr.eionet.europa.eu/mt/eu/mmr/lulucf/envxif3ca/index_html	

Country	Source for revised NFAP (links retrieved from [1])	Source for Addendum / Corrigendum / Recalculation
Netherlands	https://english.rvo.nl/sites/default/files/2019/12/National%20Forestry%20Accounting%20Plan.pdf	
Austria	https://www.bmnt.gv.at/umwelt/klimaschutz/klimapolitik_national/klimaziele_2020.html	
Poland	https://bip.mos.gov.pl/fileadmin/user_upload/bip/strategie_plany_programy/Krajowy_Plan_Rozliczen_dla_Lesnictwa/NFAP_2019_POLAND_ENG_FINAL.pdf	Recalculation (see [1]) https://www.gov.pl/attachment/b7112e1f-86dc-4dec-b95e-4f4377cbcdb0
Portugal	http://apambiente.pt/_zdata/Alteracoes_Climaticas/Mitigacao/National%20Forestry%20Accounting%20Plan_Revised%20version%20january%202020.pdf	
Romania	http://www.mmediu.ro/app/webroot/uploads/files/National%20forestry%20accounting%20plan%20of%20Romania.pdf	
Slovenia	https://www.gov.si/assets/ministrstva/MKGP/DOKUMENTI/GOZDARSTVO/NFAP_Slovenia_20191224_ang.pdf	
Slovakia	http://www.mpsr.sk/index.php?navID=1&navID2=1&sID=37&id=14987	
Finland	https://www.luke.fi/wp-content/uploads/2019/12/NFAP-for-Finland-20-December-2019.pdf	https://mmm.fi/documents/1410837/1888935/Suomen+muutokset+vertailutasoraporttiin+18062020.pdf/ece4a930-1508-8aeb-c815-d72ae2285d3b/Suomen+muutokset+vertailutasoraporttiin+18062020.pdf?t=1593079763105
Sweden	https://www.regeringen.se/48ea73/contentassets/1ef4450e8fad4c55ba0eb2f0f00366e1/national-forestry-accounting-plan-for-sweden.pdf	

Country	Source for revised NFAP (links retrieved from [1])	Source for Addendum / Corrigendum / Recalculation
United Kingdom	https://assets.publishing.service.gov.uk/government/uploads/system/uploads/attach ment_data/file/862880/national-forestry-accounting-plan-2020.pdf	

Table S3: Description of the degrees of fulfilment of principles, criteria and elements.

Degree of fulfilment	Description	Example(s)	
		Accuracy aspects	Transparency* aspects
Low fulfilment	• There are serious methodological flaws<br>• The information is not adequate, in terms of robustness, internal consistency and transparency	The FRL does not incorporate emissions and removals from HWP and dead wood pool (mandatory) or any other relevant emissions or removals reported in the GHG inventory.	Explicit information on forest management practices (FMPs) missing
Medium fulfilment	• There are no methodological flaws but major issues on specific aspects<br>• The information is in general adequate but some very specific aspects on robustness, internal consistency or transparency are lacking	Harvest intensity is based on data that only partly cover the period 2000-2009.	Missing information on some dynamic age/size-related forest characteristics
High fulfilment	• There are no methodological flaws<br>• The information is adequate	The model is calibrated, validated and robust, as well as incorporates the effects of management as in the period 2000-2009 and age/size-related forest dynamics on carbon sink.<br><br>The model is able to simulate consistent removals or emissions across required carbon pools, and	Detailed information on the evolution of harvest disaggregated between energy and non-energy uses is provided

		is consistent with the GHGI to reproduce historical data.	
* transparency issue is a first-hand assessment and therefore may hide some accuracy issues			

Table S4: Guidance table for assessing the degree of fulfilment of NFAPs to the LULUCF Regulation.

Regulation (EU) 2018/841 (LULUCF Regulation) (see Table 1)		Corresponding thematic cluster(s) (see Table 1 in the main text for more details)	Assessment keys (i.e. aspects to look at for the assessment) (main guidance sources: [3–5])
Article 8(5) (principles for setting the FRL)	Subparagraph 1	PRACTICES	• Forest management practices (FMP)<br>• Dynamic-age related forest characteristics<br>• Reference period 2000-2009 (RP)<br>• Best available data (documentation of FMP in the RP)
	Subparagraph 2	HARVEST	• Long-term forest carbon sink vs. harvest intensity (core of FMP)
	Subparagraph 3	LULUCF Inventory	• Consistency with the greenhouse gas inventory (GHGI) (1)
Annex IV.A (criteria to define the FRL)	(a) (2)	HARVEST	• Consistency with achieving long-term forest carbon sink (3)
	(b)	PRACTICES	• Accounting based on net changes in C stocks
	(c)	PRACTICES	• All emissions and removals accounted (e.g. use of biomass for energy)
	(d)	PRACTICES	• Inclusion of the Harvested Wood Products (HWP) in the FRL<br>• Comparison between FRL including HWP and FRL assuming Instantaneous Oxidation (IO)
	(e)	HARVEST	• Constant ratio between solid and energy use as in the RP
	(f)	PRACTICES	• Consideration of biodiversity in the determination of the FRL

	(g) [2]		HARVEST	• Consistency with the national projections [4]
	(h)		LULUCF Inventory	• Consistency with the GHGI (see also art. 8(5) subparagraph 3) [1,5]<br>• Model capacity to reproduce historical data (model output vs. historical time series) (see also Annex IV.B(e-ii))
Annex IV.B (main elements of the NFAP)	(a)		PRACTICES	• Description of the FRL, including the consideration of the criteria above
	(b)		LULUCF Inventory	• Considered pools and gases (reasons for those omitted)
	(c)		PRACTICES	• Description of the methodology adopted<br>• Description of FMPs and harvest intensity<br>• Description of adopted national policies
	(d)		HARVEST	• Info on future harvesting rates in different policy scenarios
	(e)	(i)	LULUCF Inventory	• Description of the area under forest management (consistently with Annex IV.A(h)), including the approach used for its development over time (static or dynamic)
		(ii)	LULUCF Inventory	• Info on historical emissions and removals from forests and HWP
		(iii)	PRACTICES	• Info on forest characteristics and their dynamics, increments, and other characteristics of FMPs as documented in the RP
		(iv)	HARVEST	• Historical and future harvesting rates disaggregated between energy and non-energy uses
(1) The year of submission of the GHGI used as reference depends on data used in the NFAP by MS.				

(2) Despite what the label suggests, the HARVEST cluster also comprises criteria related to maintaining or strengthening the forest carbon sink without constraining harvest intensity, i.e. the assessment of the comparisons between FRL and long term projections of GHG emissions and removals for the forest sector is included.

(3) Qualitative information until 2050 on the consistency of modelled emissions and removals with the long-term strategy under Regulation (EU) 2018/1999 (Governance Regulation; GR).

(4) Qualitative and quantitative information of the consistency of modelled emissions and removals with national projections reported under Regulation (EU) 525/2013

(5) Main historical data to consider: area (see also Annex IV.B(e-i)), pools and gases (see also Annex IV.B(b)), input data (e.g. same Emission Factors as in the GHGI), and modelling approach (e.g. same or similar model as in the GHGI).

Table S5: Number of NFAPs meeting degrees of fulfilment for requirements (principles, criteria and elements) of the LULUCF Regulation. Colours identify different clusters (defined in Table 1): yellow for PRACTICES, blue for HARVEST, and green for LULUCF Inventory.

Degree of fulfilment	Article 8.5			Annex IV.A								Annex IV.B							
	para 1	para 2	para 3	a	b	c	d	e	f	g	h	a	b	c	d	ei	eii	eiii	eiv
High	21	26	0	15	28	21	26	25	21	16	0	25	12	15	19	13	16	11	14
Medium	3	2	24	4	0	3	2	1	7	5	26	3	0	13	2	0	10	17	13
Low	4	0	4	9	0	4	0	0	0	6	2	0	16	0	7	13	2	0	0
Information not available	0	0	0	0	0	0	0	2	0	1	0	0	0	0	0	2	0	0	1

Table S6: relative distribution in the number of NFAPs according to the assigned adequacy (see Table 2 in the main text).

	Adequacy type		
	AGE	MANAGEMENT	POOLS
Highly adequate	14	26	5
Adequate	8	0	10
Partly adequate	6	2	13

Table S7: overview of p-values resulting from Fisher's exact test for the degree of fulfilment (by cluster; see Table 1 in the main text).

	PRACTICES	HARVEST	LULUCF Inventory
PRACTICES		2.10E-04	2.55E-19
HARVEST	2.10E-04		1.75E-11
LULUCF Inventory	2.55E-19	1.75E-11	

Table S8: overview of p-values resulting from Fisher's exact test for the model adequacy (by type of adequacy; see Table 2 in the main text).

Rate	AGE<br>Model adequacy 1	MANAGEMENT<br>Model adequacy 2	POOLS<br>Model adequacy 3
AGE<br>Model adequacy 1		4.28E-04	2.98E-02
MANAGEMENT<br>Model adequacy 2	4.28E-04		6.87E-09
POOLS<br>Model adequacy 3	2.98E-02	6.87E-09	

Table S9: overview of p-values resulting from Fisher's exact test for paired principles, criteria and elements of the LULUCF Regulation. Significance threshold: 0.05.

		Article 8.5			Annex IV.A								Annex IV.B							
		para.1	para.2	para.3	a	b	c	d	e	f	g	h	a	b	c	d	e-i	e-ii	e-iii	e-iv
Article 8.5	para. 1		0.118	0.000	0.234	0.010	1.000	0.118	0.051	0.056	0.508	0.000	0.167	0.001	0.003	0.675	0.005	0.083	0.000	0.002
	para. 2	0.118		0.000	0.000	0.491	0.118	1.000	0.611	0.143	0.004	0.000	1.000	0.000	0.002	0.013	0.000	0.005	0.000	0.001
	para. 3	0.000	0.000		0.000	0.000	0.000	0.000	0.000	0.000	0.000	0.669	0.000	0.000	0.000	0.000	0.000	0.000	0.000	0.000
Annex IV.A	a	0.234	0.000	0.000		0.000	0.234	0.000	0.000	0.003	0.753	0.000	0.001	0.036	0.001	0.574	0.062	0.032	0.000	0.001
	b	0.010	0.491	0.000	0.000		0.010	0.491	0.236	0.010	0.000	0.000	0.236	0.000	0.000	0.002	0.000	0.000	0.000	0.000
	c	1.000	0.118	0.000	0.234	0.010		0.118	0.051	0.056	0.508	0.000	0.167	0.001	0.003	0.675	0.005	0.083	0.000	0.002
	d	0.118	1.000	0.000	0.000	0.491	0.118		0.611	0.143	0.004	0.000	1.000	0.000	0.002	0.013	0.000	0.005	0.000	0.001
	e	0.051	0.611	0.000	0.000	0.236	0.051	0.611		0.028	0.005	0.000	0.357	0.000	0.000	0.005	0.000	0.001	0.000	0.000
	f	0.056	0.143	0.000	0.003	0.010	0.056	0.143	0.028		0.033	0.000	0.295	0.000	0.162	0.005	0.000	0.227	0.014	0.097
	g	0.508	0.004	0.000	0.753	0.000	0.508	0.004	0.005	0.033		0.000	0.011	0.005	0.005	0.531	0.028	0.178	0.001	0.009
	h	0.000	0.000	0.669	0.000	0.000	0.000	0.000	0.000	0.000	0.000		0.000	0.000	0.000	0.000	0.000	0.000	0.000	0.000
Annex IV.B	a	0.167	1.000	0.000	0.001	0.236	0.167	1.000	0.357	0.295	0.011	0.000		0.000	0.007	0.011	0.000	0.017	0.000	0.003
	b	0.001	0.000	0.000	0.036	0.000	0.001	0.000	0.000	0.000	0.005	0.000	0.000		0.000	0.018	0.525	0.000	0.000	0.000
	c	0.003	0.002	0.000	0.001	0.000	0.003	0.002	0.000	0.162	0.005	0.000	0.007	0.000		0.000	0.000	0.429	0.422	1.000
	d	0.675	0.013	0.000	0.574	0.002	0.675	0.013	0.005	0.005	0.531	0.000	0.011	0.018	0.000		0.057	0.014	0.000	0.000
	e-i	0.005	0.000	0.000	0.062	0.000	0.005	0.000	0.000	0.000	0.028	0.000	0.000	0.525	0.000	0.057		0.000	0.000	0.000
	e-ii	0.083	0.005	0.000	0.032	0.000	0.083	0.005	0.001	0.227	0.178	0.000	0.017	0.000	0.429	0.014	0.000		0.099	0.374
	e-iii	0.000	0.000	0.000	0.000	0.000	0.000	0.000	0.000	0.014	0.001	0.000	0.000	0.000	0.422	0.000	0.000	0.099		0.422
	e-iv	0.002	0.001	0.000	0.001	0.000	0.002	0.001	0.000	0.097	0.009	0.000	0.003	0.000	1.000	0.000	0.000	0.374	0.422	

Table S10: List of the modelling approaches used to determine the FRL and related characteristics in terms of model functioning, input and output parameters.

Country	Model name	Model type	Model subtype	Main source(s)	Scale	Proxy for the age-related forest characteristics
Belgium	SIMREG	Empirical (probabilistic)	Individual-tree distance independent	[6]	Individual tree / stand / species cohort	DBH
Bulgaria	Ad hoc FRL model	Empirical (deterministic)	Yield table driven	NFAP (p. 57)	species cohort	AGE
Czech Republic	CBM-CFS3 model	Empirical (deterministic)	Yield and growth table driven	[7]	stand / species cohort	AGE
Denmark	Ad hoc FRL model	Empirical (Probabilistic)	Individual tree survival model	NFAP (Annex 9). Database and assumptions published on line.	Whole country unique strata	DBH
Germany	Ad hoc FRL model & Yasso15 model	Empirical (deterministic)	Yield table driven	N/A	forest types	OTHER (Volume)
Estonia	Ad hoc FRL model	Empirical (deterministic)	Area shift matrix	NFAP (p. 25)	Stand / species cohort	AGE
Ireland	CBM-CFS3 model	Empirical (deterministic)	Yield and growth table driven	[7]	stand / species cohort	AGE
Greece	Ad hoc FRL model	Empirical (deterministic)	Increment to volume model	NFAP (section 3.3)	Administrative regions/stand / species cohort	OTHER (biomass-increment ratio)
Spain	Ad hoc FRL model (i.e. Vael model)	Empirical (probabilistic)	Size class matrix	NFAP (p. 29)	Region, species cohort	diametric class and AGE
France	MARGOT model	Empirical (probabilistic)	Size class and disappearance matrix	[8]	forest strata	DBH class and basal area class

Country	Model name	Model type	Model subtype	Main source(s)	Scale	Proxy for the age-related forest characteristics
Croatia	Ad hoc FRL model (i.e.HS-MODEL)	Empirical (deterministic)	Yield tables	NFAP (p. 52)	stand / species cohort	AGE
Italy	for-est model	Hybrid (empirical + mechanistic)	Yield table	[9]	forest type	OTHER (biomass density)
Cyprus	Ad hoc FRL model	Empirical (deterministic)	GHG data driven	NFAP (p. 21)	forest type	OTHER (average increment)
Latvia	AGM, Yasso & EPIM models	Empirical (probabilistic)	Data driven	[10]	forest element (species cohort)	AGE and DBH
Lithuania	EFDM model	Empirical (probabilistic)	Area-volume based matrix	[11]	forest type	VOLUME-AREA
Luxembourg	Ad hoc FRL model	Empirical (deterministic)	Area-based development	NFAP (p. 11)	forest type	AGE
Hungary	CASMOFOR model	Empirical (deterministic)	Yield table driven	[12]	species group / forest type	AGE
Malta	Ad hoc FRL-model	Empirical (deterministic)	Data driven	NFAP (p. 40)	forest type	OTHER (average increment)
Netherlands	EFISCEN space	Empirical (probabilistic)	Individual tree	Description of the model not available yet.<br><br>A study using EFISCEN Space is [13]	plot	SIZE (DBH)
Austria	CALDIS-VB & Yasso model	Empirical (probabilistic)	Individual-tree distance independent	[14]	stand	DBH
Poland	CBM-CFS3 model and other ancillary approaches	Empirical (deterministic)	Yield and growth table driven	[7]	stand / species cohort	AGE

Country	Model name	Model type	Model subtype	Main source(s)	Scale	Proxy for the age-related forest characteristics
Portugal	Ad-hoc FRL model	Empirical (deterministic)	Historical GHG data driven	NFAP (p. 70)	N/A	OTHER (average increment)
Romania	Ad hoc FRL model	Empirical (deterministic)	Yield table driven	NFAP (p. 24)	species group / forest type	AGE
Slovenia	Ad hoc FRL model	Empirical (deterministic)	Yield and growth table driven	NFAP (p. 21)	country aggregated	DBH
Slovakia	Ad hoc FRL model	Empirical (deterministic)	Yield table driven	NFAP (p. 35)	species group	AGE
Finland	MELA and Yasso07 model	Empirical (probabilistic)	Individual-tree distance independent	[15]	individual tree / plot	AREA and DBH
Sweden	Heureka Reg Vis model & Q-model	Empirical (probabilistic)	Individual-tree distance independent	[16] Other information available at ([1])	individual tree / management unit	AGE, SIZE
United Kingdom	CARBINE model	Empirical (deterministic)	Yield table driven	([2])	species group	AGE

[1] https://www.slu.se/en/departments/forest-resource-management/program-project/forest-sustainability-analysis/heureka/heureka-systemet/regwise-/

[2] https://www.forestresearch.gov.uk/research/forestry-and-climate-change-mitigation/carbon-accounting/forest-carbon-dynamics-the-carbine-carbon-accounting-model/

Table S.10 (continued)

Country	Input data	Period (input data)
Belgium	RFI, GHGI, FAO, other studies	1994-2016, FAO 2000-2009
Bulgaria	FMP Forest management plans, RF Forestry fund reporting (table 3), other doc. for 2000-2009	RF data for 2000, 2005, 2010
Czech Republic	Stand -wise forest inventory, GHGI, Cadastre office COSMC, Forest act 289/1995	2004 used to calculate data for 2000-2009 (growing stock and increment); 2010 for initial state of forests for the FRL projection (p. 12-18). FMP from 2000-2009 (p. 20-22).
Denmark	NFI, FAOSTAT, Danish wood industry questionnaire	NFI 2002-2017, FAO 2018
Germany	NFI, Inventory Study, Forest Soil Inventories, Forest statistics	2002; 2008; 1987-1994; 2004-2008; 2001-2017
Estonia	NFI, GHGI, other sources of information such as FAOSTAT for HWP	2000-2009 for data concerning management practices and stratification. NFI 2017 data to define the state of forest (see section 3.3).
Ireland	NFI, GHGI, FAO/Eurostat, other studies	2006-2017
Greece	NFI (stand-wise), GHGI, Statistical (nationally aggregate) data, IPCC Gain loss approach	2009 values are used for the stratification and area of forest land (p.20). Annual harvest ratio for FMPs are defined based on 2000-2009 data (p.28)
Spain	NFI, FAOSTAT	NFI2 (1986-1996), NFI3 (1997-2007) and NFI4 (2007-currently under development
France	NFI, forestry extraction survey (AGRESTE)	NFI harvest rates (2005-2014; 2003-2009)
Croatia	NFI, GHGI, HS-FOND - state database of individual forest management plans (p. 50)	2016 state of the forest is used as calibration (p. 50).
Italy	NFI, ISTAT, IPCC, other projects and studies.	1985-2005. Following years for other information than NFIs.
Cyprus	NFI, NIR, other data sources for area	2011-2012, 2001-2011

Country	Input data	Period (input data)
Latvia	NFI, GHGI. See Fig. 13 on p. 38.	2004-2008; calculated backwards to 2000 and forward to 2009. See fig. 37.
Lithuania	NFI	2000-2009
Luxembourg	NFI, GHGI (for areas) ANF-Administration de la Nature et des Forêts), for HWP FAO and comex database	2000-2010, 1960-2018 for FAO
Hungary	National Forestry Database (NFDB), NFI, GHGI 2020, CASMOFOR	2000-2009; 2010-2017 (DW)
Malta	Documents and maps by different national authorities and associations; functions for modelling from various compatible international sources	various sources from 2000-2014 described in more detail
Netherlands	NFI, other studies (i.e. diameter increment models for prevailing tree species (p.42) and mortality probabilities (P. 49))	NFI-5 (2001-2005) and NFI-6 (2012-2013). Netherland reports that there is methodological inconsistencies in the FAO statistics on wood fuel harvests from 2015 onwards that requires a revision of the methodology as used in the NIR (see section 3.2.1 and Appendix 2)
Austria	NFI, GHGI	2000-2009
Poland	NFI (WISL), Forest DataBank (FDB), National Statistics (GUS)	WISL (2006-2010) (p. 28); GUS (2010-2017)
Portugal	NIR	NIR 2018 (2000-2009)
Romania	NIR, NFI, MEWP, yield tables, IPCC 2006 gl	NIR 2019, NFI 2008-2012
Slovenia	GHGI, NFIs 2000, 2007, 2012 and 2018, FAOSTAT (integrated)	2000-2012, 1900-now (HWP)
Slovakia	GHG NIR 2016, NFIM, Green reports, FAO, Central forestry databases and forest management plans	NFIM 2005 and 2015, Green reports 2000-2009
Finland	NFI, GHGI, Forest management guidelines 2006, Forest act 224/1997	FMP from 2000-2009; state of forests from 2009-2015. See NFAP (p. 23-24).

Country	Input data	Period (input data)
Sweden	NFI, GHGI. See Fig. 12 on p. 34.	LB and Org. soils: 2008-2012; Mineral soils, HWP and other emissions: 2000-2009.
United Kingdom	NFI, GHGI, other studies. See table 3.1 on p. 22.	1996-2017. The UK reports that the data sources have been adjusted for a consistent reporting year of 2011 for the FRL.

Table S.10 (continued)

Country	Definition of harvest intensity within forest management practices	Outcome type	C pools other than living biomass as included in the modelling outcome
Belgium	FMP are probabilistic/ Harvest probabilities by strata or age class.	VOLUME	NO
Bulgaria	FMP deterministic (aggregated on stands available for thinning and final cut). Harvest volume per growing stock.	AREA and VOLUME	NO
Czech Republic	Harvest volume per growing stock	VOLUME and AREA	C stock for all C pools
Denmark	Harvest probabilities by strata and age class	CARBON STOCK IN BIOMASS	NO
Germany	Harvest probabilities by strata or age class	AREA and volume classes	Aggregated soils C stock
Estonia	Harvest area per area available for harvest (as the proxy for FPMs)	AREA and VOLUME	Dead wood
Ireland	Harvest per increment (as the proxy for FPMs)	VOLUME and AREA	C stock for all C pools
Greece	Ratio of harvest per increment (as the proxy for FPMs)	INCREMENT per VOLUME	Dead wood
Spain	Harvest biomass per biomass in the growing stock	C stock in Biomass	NO
France	Extraction rate per diameter class	C stock in biomass	NO
Croatia	Harvest volume per growing stock	AREA, Volume and Increment	NO
Italy	Harvest volume per growing stock	BIOMASS DENSITY AND INCREMENT	NO
Cyprus	Harvest volume per hectare	NOT PROVIDED	NO
Latvia	Harvest volume probability per area	AREA, Volume and Increment	Aggregated soils C stock
Lithuania	Harvest probabilities by strata or age class	AREA, Volume and Increment	Dead wood
Luxembo urg	Harvest volume per growing stock	AREA, Volume and increment	Dead Wood
Hungary	Harvest volume per growing stock	AREA, VOLUME	Dead wood
Malta	No harvest	NOT PROVIDED	NO
Netherlan ds	Harvest probabilities by strata or age class	Area, VOLUME	Dead Wood simple balance model

Country	Definition of harvest intensity within forest management practices	Outcome type	C pools other than living biomass as included in the modelling outcome
Austria	Harvest probabilities by strata	AREA, volume and increment	Aggregated soils C stock
Poland	Harvest volume per growing stock	AREA and VOLUME	C stock for all C pools
Portugal	Harvest volume per hectare	NOT PROVIDED	NO
Romania	Harvest volume per growing stock	VOLUME	NO
Slovenia	Harvest volume per growing stock	VOLUME	NO
Slovakia	Harvest volume per growing stock	VOLUME	NO
Finland	Harvest area per area available for harvest, constrained by minimum DBH	AREA, VOLUME	Aggregated soils C stock
Sweden	Harvest per increment	VOLUME	All C pools are included
United Kingdom	Harvest area per area available for harvest	AREA	YES

Table S11: Overview of the consistency about carbon pools and CO2 and non-CO2 gases between NFAPs and GHG inventories (source: [1]). Values represent the number of countries.

Type of consistency / inconsistency between NFAP and GHG inventory	Living biomass (1)	Deadwood (1)	HWP (2)	Litter (1)	Mineral Soil Organic Carbon (1)	Organic Soil (1)	Nitrogen Fertilization (3)	Drainage and rewetting (4)			Mineralization (5)	Biomass burning (6)		
	CO_2						N_2O	CO_2	CH_4	N_2O	N_2O	CO_2	CH_4	N_2O
Estimated in NFAP and GHGI	27	16	27	7	9	10	2	1	7	9	1	7	18	18
Not estimated in NFAP but estimated in GHGI				1	2	1		1		1	1	9	8	8
Estimated in NFAP but not in GHGI	1	3		2								3		
Estimate could be included in another pool or reporting table		1	1	1				2						
Not occurring / Not assessed / Included elsewhere		8		17	17	17	26	24	21	18	26	9	2	2
References to Common Reporting Format (CRF) Tables in the GHG inventory: (1) Table 4.A; (2) Table 4.Gs1; (3) Table 4 (I); (4) Table 4 (II); (5) Table 4 (III); (6) Table 4 (V).														

References

1. EC - European Commission. Commission Staff Working Document Assessment of the Revised National Forestry Accounting Plans 2021–2025 Accompanying the document Commission Delegated Regulation amending Annex IV to Regulation (EU) 2018/841 of the European Parliament and of the Council as regards the forest reference levels to be applied by the Member States for the period 2021-2025. 2020. https://eur-lex.europa.eu/legal-content/EN/TXT/?uri=CELEX:52020SC0236

2. Korosuo A, Vizzarri M, Pilli R, Fiorese G, Colditz R, Abad Viñas R, et al. Forest reference levels under Regulation (EU) 2018/841 for the period 2021-2025. Luxembourg: Publications Office of the European Union; 2021.

3. Forsell N, Korosuo A, Federici S, Gusti M, Rincón-Cristóbal J-J, Rüter S, et al. Guidance on developing and reporting Forest Reference Levels in accordance with Regulation (EU) 2018/841. Luxembourg: Publications Office of the European Union; 2018.

4. EC - European Commission. Commission Staff Working Document Assessment of the National Forestry Accounting Plans Regulation (EU) 2018/841 of the European Parliament and of the Council on the inclusion of greenhouse gas emissions and removals from land use, land use change and forestry in the 2030 climate and energy framework, and amending Regulation(EU)No 525/2013 and Decision No 529/2013/EU Accompanying the document Communication from the Commission to the European Parliament, the Council, the European Economic and Social Committee and the Committee of the Regions United in delivering the Energy Union and Climate Action - Setting the foundations for a successful clean energy transition. 2019. https://eur-lex.europa.eu/legalcontent/NL/TXT/?uri=CELEX:52019SC0213

5. IPCC. Refinement to the 2006 IPCC Guidelines for National Greenhouse Gas Inventories, vol 4. Agriculture, Forestry and Other Land Use. Switzerland; 2019. https://www.ipcc-nggip.iges.or.jp/public/2019rf/vol4.html

6. Perin J, Pitchugin M, Hébert J, Brostaux Y, Lejeune P, Ligot G. SIMREG, a tree-level distance-independent model to simulate forest dynamics and management from national forest inventory (NFI) data. Ecol Modell. 2021;440:109382.

7. Kurz WA, Dymond CC, White TM, Stinson G, Shaw CH, Rampley GJ, et al. CBM-CFS3: A model of carbon-dynamics in forestry and land-use change implementing IPCC standards. Ecol Modell. 2009;220:480–504.

8. Colin A, Wernsdörfer H, Thivolle-Cazat, A. Bontemps J-D. France. In: Barreiro S, Schelhaas M-J, McRoberts RE, Kändler G, editors. For Invent Proj Syst Wood Biomass Availab. Springer International Publishing Switzerland; 2017. p. 159–174.

9. Federici, S; Vitullo, M; Tulipano, S; De Lauretis R, Seufert G. An approach to estimate carbon stocks change in forest carbon pools under the UNFCCC : the Italian case. iForest - Biogeosciences For. SISEF - Italian Society of Silviculture and Forest Ecology; 2008;1:86–95.

10. Šņepsts G, Kārkliņa I, Lupiķis A, Butlers A, Bārdule A, Lazdiņš A. AGM model description (Draf No. 2018-01–1). 2018.

11. Packalen T, Sallnäs O, Sirkiä S, Korhonen K, Salminen O, Vidal C, et al. The European Forestry Dynamics Model: Concept, design and results of first case studies. Luxembourg: Publications Office of the European Union; 2014.

12. Somogyi Z, Hidy D, Gelybó G, Barcza Z, Churkina G, Haszpra L, et al. Modeling of biosphere atmosphere exchange of greenhouse gases Models and their adaptation. In: Haszpra L, editor. Atmos Greenh Gases Hungarian Perspect. 2010. p. 201–28.

13. Nabuurs GJ, Schelhaas M, Oldenburger J, de Jong A, Schrijver RAM, Woltjer GB, et al. Nederlands bosbeheer en bos- en houtsector in de bio-economie : scenario’s tot 2030 in een internationaal bio-economie perspectief. Wageningen; 2016.

14. Ledermann T, Kindermann G, Gschwantner T. National Woody Biomass Projection Systems Based on Forest Inventory in Austria. In: Barreiro S, Schelhaas MJ, McRoberts RE, Kändler G, editors. For Invent Proj Syst Wood Biomass Availab. Springer International Publishing, Switzerland; 2017. p. 79–95.

15. Hirvelä H, Härkönen K, Lempinen R, Salminen O. MELA2016 : Reference Manual. Helsinki, Finland; 2017.

16. Wikström P, Edenius L, Elfving B, Eriksson LO, Lämås T, Sonesson J, et al. The Heureka Forestry Decision Support System: An Overview. Math Comput For & Nat Sci (MCFNS); Vol 3, No 2 MCFNS August 28, 2011. 2011.
