# Supplementary material for: Setting the forest reference levels in the European Union: overview and challenges
Source: Carbon Balance Manag. 2021 Jul 31;16:23. doi: 10.1186/s13021-021-00185-4 (PMC8325867; doi:10.1186/s13021-021-00185-4)
Supplement: Supplementary file 2 — Additional file 2. Supplementary figure supporting the harvest-biomass sink assessment results. [file 13021_2021_185_MOESM2_ESM.pdf]

Additional file 2 – Supplementary Figures

Setting the Forest Reference Levels in the European Union: overview and challenges

Matteo Vizzarri, Roberto Pilli, Anu Korosuo, Viorel NB Blujdea, Simone Rossi, Giulia Fiorese, Raul Abad-Viñas, Rene Colditz, and Giacomo Grassi

Figure S1: Relative share of even-aged forest area (on the y-axis) distinguished between 11 age classes (on the x-axis) with age span of 20 years, as reported by the Carbon Budget Model for the year 2010 [1]. For Greece and Spain, where the forest area is mostly classified as uneven-aged, no data is reported. No data is available for Malta..2

Figure S1: Relative share of even-aged forest area (on the y-axis) distinguished between 11 age classes (on the x-axis) with age span of 20 years, as reported by the Carbon Budget Model for the year 2010 [1]. For Greece and Spain, where the forest area is mostly classified as uneven-aged, no data is reported. No data is available for Malta.

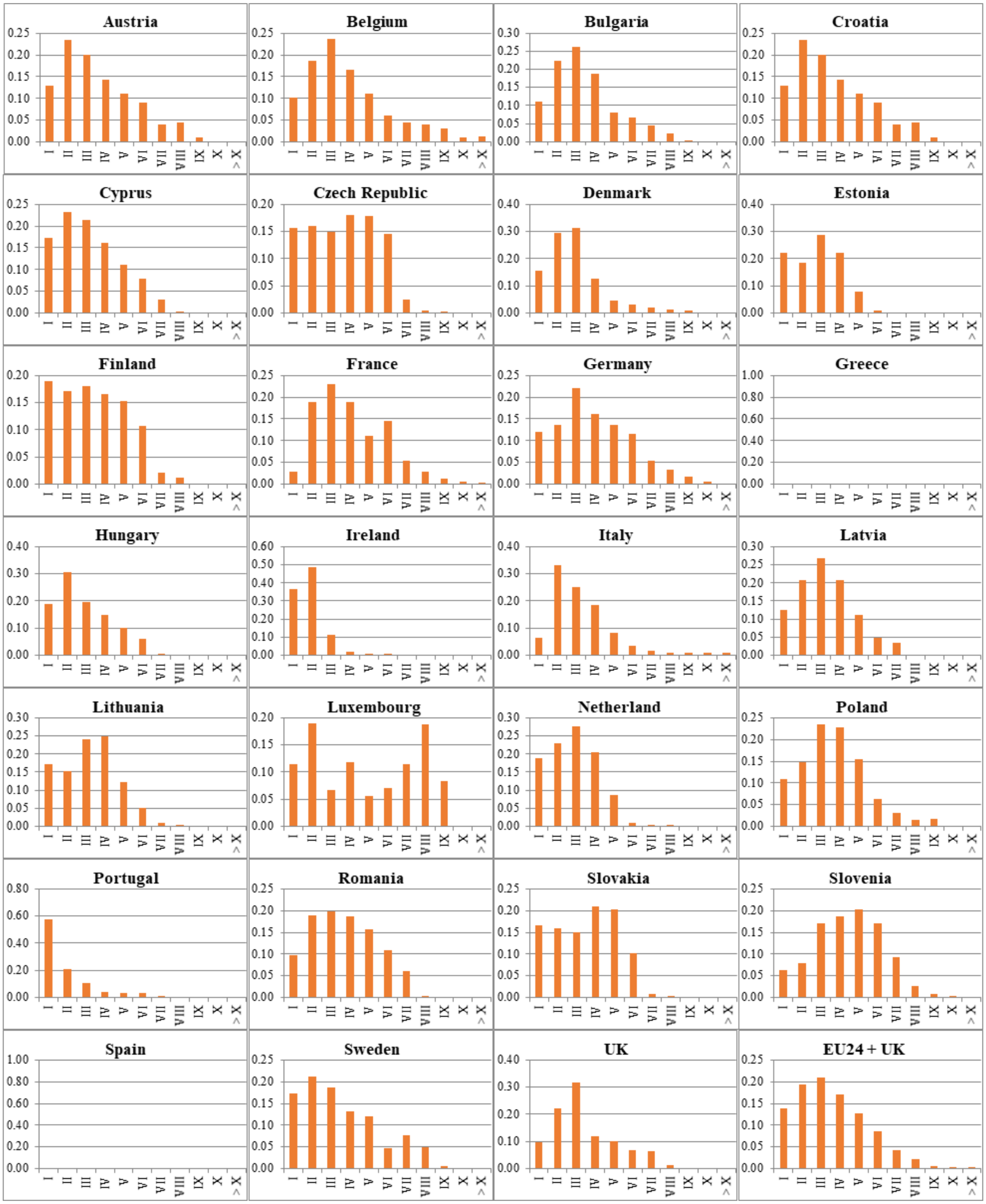

References

1. Pilli R, Grassi G, Kurz WA, Viñas RA, Guerrero NH. Modelling forest carbon stock changes as affected by harvest and natural disturbances. I. Comparison with countries' estimates for forest management. Carbon Balance Manag. 2016;11:5.
